# A Natural product-based composite nanozyme synergistically induces ferroptosis for lung cancer therapy

**DOI:** 10.1039/d6ra00383d

**Published:** 2026-05-26

**Authors:** Yishun Jin, Chen Huang, Zhenbo Geng, Huang Li, Huiqing Ge, Pengfei Wang, Chaojun Dai, Jun Li, Xiaohua Yan, Chen Li, Yan Zheng

**Affiliations:** a Department of Traditional Chinese Medicine, Fuzhou University Affiliated Provincial Hospital Fuzhou 350001 China yanxiaohua@fjsl.com.cn; b Department of Thoracic Surgery, Fuzhou University Affiliated Provincial Hospital Fuzhou China; c School of Pharmacy, Fujian University of Traditional Chinese Medicine Fuzhou 350122 China; d Department of Critical Care Medicine, Fuzhou University Affiliated Provincial Hospital, Fujian Provincial Hospital Fuzhou 350001 China; e Fujian Provincial Center for Critical Care Medicine, Fujian Provincial Key Laboratory of Critical Care Medicine Fuzhou 350001 China; f College of Pharmacy, Hainan Academy of Medical Sciences, Hainan Medical University Haikou Hainan 571199 China hy0211100@muhn.edu.cn; g Department of Oncology, Fuzhou University Affiliated Provincial Hospital China zhengyan0996@fzu.edu.cn

## Abstract

The treatment of lung cancer remains a significant clinical challenge. Inducing ferroptosis is a promising therapeutic strategy to overcome treatment resistance in lung cancer. Herein, we report a composite nanozyme, Fe@Arc, engineered by coordinating arctigenin with iron ions followed by a high-temperature carbonization. This fabrication method yielded a nanozyme with a high drug-loading capacity and controlled release kinetics. Following intravenous administration, Fe@Arc efficiently accumulated at the tumor site owing to its enhanced permeability and retention effect. In the tumor site, it exerted a potent anti-tumor effect by catalyzing the Fenton reaction and releasing arctigenin, which synergistically triggered ferroptosis in cancer cells. This work presents a multifaceted platform that not only overcomes the pharmacological limitations of herbal extracts but also establishes a robust framework for developing novel ferroptosis inducers, demonstrating significant potential for clinical translation.

## Abstract 2

The treatment of lung cancer remains a significant clinical challenge.

## Graphical abstract

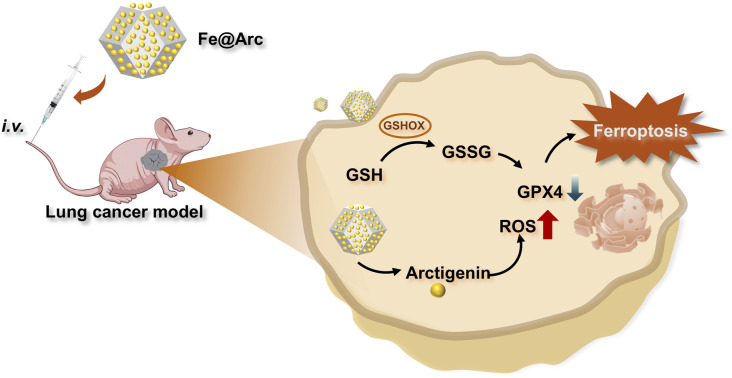

## 1 Introduction

Lung cancer remains the leading cause of tumor-related mortality globally, inflicting a substantial burden on public health systems.^1,2^ Histologically, it is primarily classified into non-small cell lung cancer (NSCLC) and small cell lung cancer (SCLC), with NSCLC accounting for approximately 85% of cases.^3^ The pathogenesis of lung cancer is a complex process shaped by genetic susceptibility and environmental exposures, such as tobacco smoking.^4^ However, the epidemiological landscape has shifted recently, with a rising incidence in never-smokers, particularly in the East Asian populations. At the molecular level, the discovery of diverse somatic genetic alterations has laid the groundwork for targeted therapies, which have significantly improved patient outcomes.

Ferroptosis, a unique iron-dependent form of programmed cell death (PCD) that is morphologically, biochemically, and genetically distinct from other PCD subtypes, is characterized by the irreversible accumulation of lipid peroxides in cellular membranes, driven by excessive reactive oxygen species (ROS) generation and impairment of the glutathione (GSH)-glutathione peroxidase 4 (GPX4) antioxidant system.^5–10^ The identification of ferroptosis has shed fresh light on the regulatory mechanisms underlying cellular demise while unlocking innovative therapeutic strategies for targeting malignant cells. Unlike normal somatic cells, cancer cells typically display dysregulated metabolic pathways (*e.g.*, enhanced iron uptake and altered lipid metabolism) and a heightened susceptibility to ferroptosis induction—two traits that make them particularly responsive to treatments designed to trigger this unique form of programmed cell death.^11–13^ Preclinical investigations have shown that inducing ferroptosis enables the selective elimination of cancer cells while preserving normal somatic cells—a characteristic that underscores its promise as a precision-focused cancer therapeutic approach. Beyond this, the aberrant regulation of ferroptosis-associated signalling cascades—specifically those governing iron homeostasis, lipid biosynthesis, and redox-defined mechanisms—has been linked to the pathogenesis and progression of multiple cancer types, such as NSCL cancer and pancreatic ductal adenocarcinoma.^14–17^ Thus, deciphering the molecular underpinnings that govern ferroptosis progression, along with developing pharmacological compounds capable of triggering or suppressing this unique programmed cell death modality, holds tremendous significance for advancing both basic cancer biology research and clinical therapeutic development.^18–22^ Nanotechnology-based approaches leverage the unique physicochemical properties of nanomaterials, such as their small size, large surface area, and tunable surface chemistry, to overcome these challenges and develop effective therapeutic agents. Engineered nanocarriers can be functionalized with ligands that exhibit high affinity for facilitating targeted delivery and controlled release of therapeutic payloads.^23^ Techniques rooted in nanotechnology capitalize on the distinctive physicochemical traits of nanoscale therapeutic agent development—including ultra-small dimensions, expanded surface area, and adjustable surface chemistry—to address these hurdles. Nanocarriers designed through rational engineering can be modified with high-affinity ligands; these ligands not only enhance targeted delivery but also enable controlled release of the therapeutic cargo they encapsulate.^24,25^ Moreover, the enhanced permeability and retention (EPR) effect, a phenomenon characterized by the leaky vasculature and poor lymphatic drainage of tumor tissues, enables the passive accumulation of nanoparticles within the tumor microenvironment.^26^ This multiple active agent's integration strategy holds great promise for improving the therapeutic index of conventional chemotherapeutics, overcoming drug resistance, and enabling multifunctional therapeutic applications. As such, nanotechnology represents a paradigm shift in ferroptosis-related cancer treatments, offering a versatile platform for the development of next-generation anticancer modalities with enhanced precision and efficacy.^27^ Hence, the development of novel drug delivery systems will be crucial in optimizing treatment strategies and ultimately improving the overall survival rate and quality of life of patients with lung cancer.

**Fig. 1:**
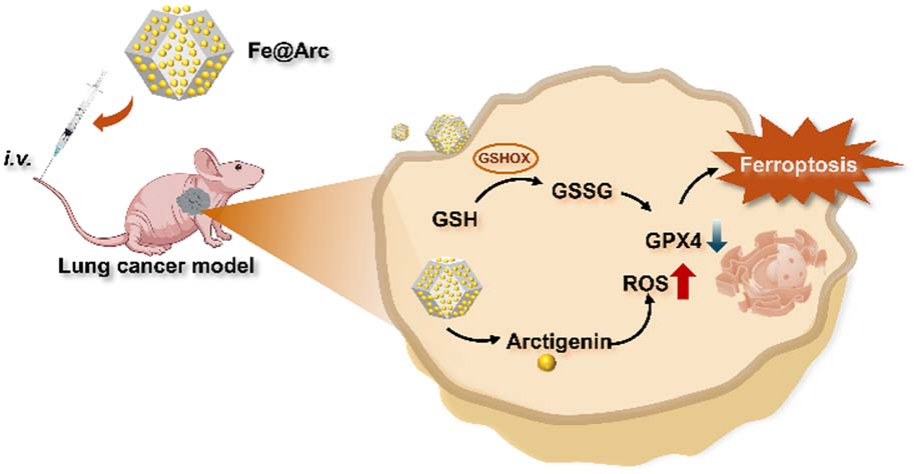
Schematic of the fabrication of the arctigenin-integrated composite nanozyme and its use in collaborative ferroptosis induction for lung cancer treatment.

In the present work, we fabricated a composite nanozyme termed Fe@Arc *via* a high-temperature carbonization method using arctigenin as the key component. Arctigenin, a natural lignan compound derived from the fruits of *Arctium lappa* L.—a medicinal herb widely used in traditional Chinese medicine—has been shown to exert remarkable anti-tumor activities. When administered intravenously in an NSCLC model, this Fe-based nanozyme triggers ferroptosis by depleting GSH in tumor cells (Fig. 1). The consequent production of glutathione disulfide (GSSG) leads to a reduction in the expression of GPX4, thereby accelerating ferroptosis, an iron-dependent form of programmed cell death. Meanwhile, the encapsulated arctigenin, a potent anti-cancer agent, exerts a synergistic effect by elevating intracellular ROS levels. Comprehensive characterization assays confirmed that this rationally engineered nanozyme features high encapsulation efficiency, favourable biosafety profiles, and robust anti-tumor activity in a murine lung tumor model. Specifically, Fe@Arc enhances ROS generation and modulates gene expression in a synergistic manner, which collectively promotes tumor ferroptosis and achieves significant tumor growth inhibition. This study reports an arctigenin-based ferroptosis modulator for the first time, offering new insights for broadening the therapeutic applications of traditional herbal extracts.

## 2 Materials and methods

### 2.1 Materials

Arctigenin was purchased from MedChemExpress. Annexin V-FITC/PI and Hifair® III One Step RT-qPCR SYBR Green Kit were purchased from Yeasen Biotechnology, Shanghai, China. Hoechst 33 342 and cell count kit-8 (CCK-8) were purchased from Solarbio. Serum-free cell freezing medium was purchased from Shenzhen Dakewe Bio-engineering Co., Ltd A549 cells were purchased from Zhejiang Meisen Cell Technology Co., Ltd (Hangzhou, China).

### 2.2 Synthesis and characterization of Fe@Arc

Fe@ZIF-8 precursors were synthesized using a host–guest strategy. 3.54 g of 2-methylimidazole and 100 mg of Fe(acac)_3_ were added to 60 mL methanol in a 200 mL flask; this was taken as solution A. 1.6 g of Zn(NO_3_)_2_·6H_2_O was dissolved in 60 mL methanol, and this was taken as solution B. Then, solution A was mixed with solution B, and the mixture was further stirred for 12 h. Subsequently, the Fe@ZIF-8 powder was meticulously collected *via* centrifugation at 12 000 rpm for 15 min, thoroughly washed multiple times with methanol to remove any residual impurities, and subjected to drying at 70 °C in a vacuum oven overnight. The Fe@ZIF-8 powder was then placed in a tubular furnace and pyrolyzed at 950 °C for 3 h in an argon atmosphere to obtain the Fe nanoparticles. Subsequently, 10 mg of Fe NPs and 10 mL of 40 mg mL^−1^ DSPE-S-S-PEG were mixed and ultrasonicated for 20 min, followed by the addition of 1.5 mL of 1 mg mL^−1^ Arc (in DMSO). The mixture was further stirred for 12 h, and the product Fe@Arc was collected by centrifugation and washed three times with PBS. Cy5-labeled Fe@Arc nanozymes were prepared by incubating Fe@Arc with Cy5-labeled DSPE-PEG. During this process, DSPE-PEG spontaneously wrapped around the surface of the nanozyme, resulting in a stable fluorescence labeling.

### 2.3 ˙OH generation by Fe NP-mediated catalytic reaction

The catalytic activity of Fe NPs was measured using TMB as a probe, which could be converted to oxidized TMB (blue color) by ˙OH. Briefly, different concentrations of Fe NPs and H_2_O_2_ (100 µM) were successively added to PBS solutions with TMB (50 µg mL^−1^), and the mixtures were shaken at 37 °C for 10 min. After centrifugation, the absorption spectra of the supernatants were measured.

Fe NPs and H_2_O_2_ (10 mM) were mixed with various MB (20 µg mL^−1^) solutions at acidic pH, and the mixtures were shaken at 37 °C for 10 min. After ultrafiltration, the ˙OH-induced MB degradation was measured by the absorbance change at 652 nm.

Electron spin resonance (ESR) analysis was carried out using DMPO as the spin trapper. To confirm the Fe NP-mediated ˙OH generation, 10 mM NaAc-HAc buffer solution (pH 4.3) containing 10 mM H_2_O_2_, Fe NPs (100 µg mL^−1^), and 100 mM DMPO was ultrasonicated for 1 min. Then, the mixture was transferred to a quartz tube for ESR measurements.

### 2.4 Cellular uptake and subcellular localization

Cells were initially seeded into 24-well plates and permitted to adhere overnight. Subsequently, these cells were incubated with Cy5-labeled Fe@Arc nanozymes for 4 hours at 37 °C to facilitate cellular uptake. The cells were then co-stained with LysoTracker and Hoechst 33 342 (Solarbio, C0031) for 30 minutes at 37 °C to visualize lysosomes and nuclei, respectively. Following staining, the staining agents were removed, and the cells were fixed with 4% paraformaldehyde for 15 minutes at room temperature to preserve cellular structures. Finally, the samples were imaged using a confocal laser scanning microscope to capture the detailed intracellular localization of the nanozymes.

### 2.5 Cellular cytotoxicity assay

A549 cells were precisely seeded into a 96-well plate and permitted to adhere overnight within an incubator. Once adhered, the initial culture medium was gently replaced with a fresh medium, which was carefully supplemented with varying concentrations of the test drugs to assess their effects. Following a 24 hour incubation period under the same controlled conditions, 100 µL of the fresh culture medium, enriched with 10% CCK-8 solution (Solarbio, CA1210), was delicately added to each well to initiate the viability assessment. The absorbance of each well was then meticulously measured at 450 nm using a high-precision microplate reader.

### 2.6 *In vivo* therapeutic effect analysis

All animal procedures were conducted in strict accordance with the guidelines approved by the Institutional Animal Care and Use Committee of Hainan Medical University (HYLL-2023-182). A549 cells, suspended at a concentration of 1 × 10^6^ cells in 100 µL of PBS, were subcutaneously injected into the right flank of each mouse to induce tumor formation. Tumor growth was meticulously monitored on a daily basis, and the tumor volume was calculated using the following formula: volume = (length × width^2^)/2. Once the tumors reached an average volume of 75 mm^3^, the mice were randomly assigned to four experimental groups, with five mice per group. The designated drugs were administered intravenously *via* the tail vein at a 3 day interval for a total treatment duration of 21 days. Throughout the treatment period, both tumor volume and body weight were measured every 3 days to assess the therapeutic response and potential side effects. At the end of the treatment period, the mice were humanely euthanized, and the tumors were carefully excised and weighed to evaluate the overall treatment efficacy.

### 2.7 Instruments

Powder X-ray diffraction (XRD) patterns were recorded using a Rigaku Miniflex-600 diffractometer. Transmission electron microscopy (TEM) was conducted using Hitachi-7700. High-angle annular dark field scanning transmission electron microscopy (HAADF-STEM) images were recorded using JEM-ARM200F (JEOL). The energy-dispersive X-ray spectroscopy (EDS) mapping was performed using JEM-2100F. X-ray photoelectron spectroscopy (XPS) was performed using a scanning X-ray microprobe (PHI 5000 Versa, ULAC-PHI). Scanning electron microscopy (SEM) images were taken with Nova NanoSEM 230. Fluorescence imaging was performed by confocal microscopy (Nikon C2). The absorption spectra were measured using an ultraviolet-visible (UV-vis) UH4150 spectrophotometer (Hitachi). Metal content was measured using an inductively coupled plasma mass spectrometer (ICP-MS, PlasmaQuad 3, Thermo Fisher Scientific). Hydrodynamic diameters and zeta potentials were determined with a Zetasizer nano ZS instrument (Malvern). Cancer cell apoptosis was monitored using a flow cytometer (CytoFLEX, Beckman).

### 2.8 Statistical analysis

Data represent the mean ± s. d. values from indicated independent replicates. Statistical analysis was conducted using GraphPad Prism. For comparisons between two groups, means were compared using unpaired two-tailed Student's *t*-test. A value of *P* < 0.05 was considered statistically significant.

## 3 Results and discussion

### 3.1 Construction of Fe@Arc

**Fig. 2:**
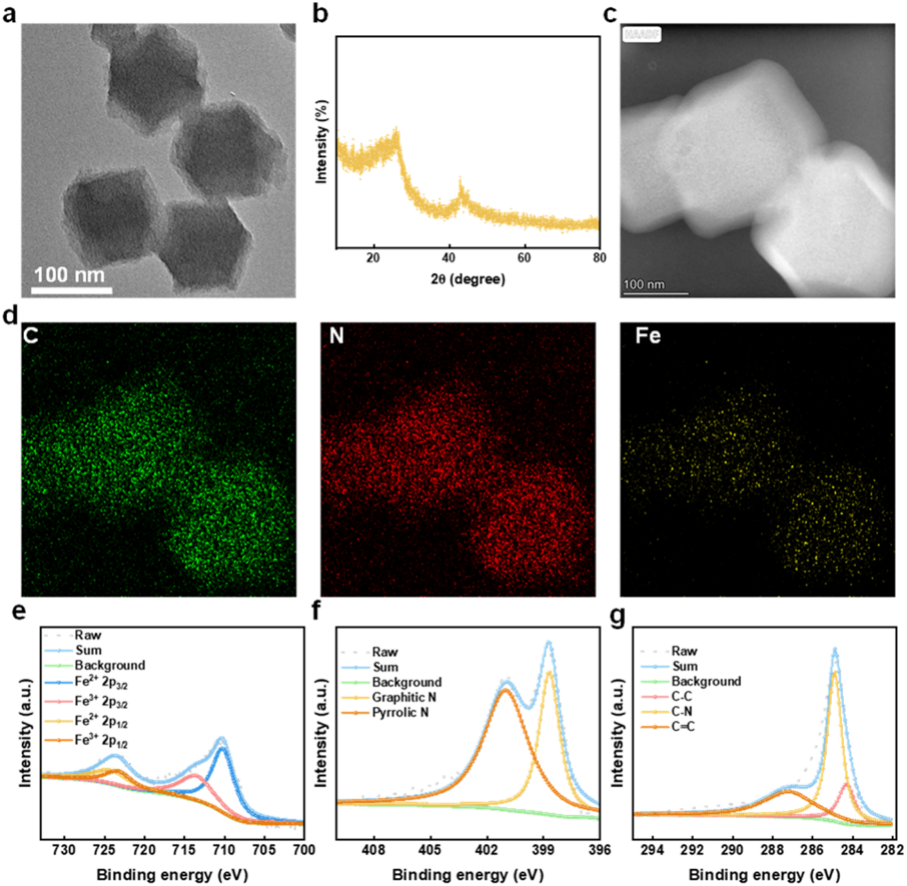
(a) TEM image of Fe@Arc scale bar = 100 nm. (b) XRD pattern of Fe@Arc. (c and d) Elemental mapping images of Fe@Arc scale bar = 100 nm. (e) Fe 2p, (f) N 1s, and (g) C 1s XPS spectra of Fe@Arc.

The nanozyme-based ferroptosis inducer Fe@Arc was constructed through a two-step process involving the high-temperature pyrolysis of the Fe@ZIF-8 precursor, followed by the loading of arctigenin *via* hydrophobic interactions. Arctigenin is a natural lignan compound. It is primarily extracted from the dried fruits of *Arctium lappa* L., commonly known as burdock. This plant has a long history in Chinese traditional medicine. Arctigenin exhibits significant pharmacological properties. These include potent anti-inflammatory and anti-tumor activities. Its mechanism often involves the modulation of key cellular signaling pathways. To begin with, we synthesized and characterized the Fe@ZIF-8 precursor. Transmission electron microscopy (TEM) analysis verified that it exhibited a homogeneous morphology, with an average particle size of around 110 ± 5.8 nm (Fig. S1). Following pyrolysis and drug loading, the resulting Fe@Arc nanozyme maintained a uniform and regular spherical morphology without significant aggregation, as depicted in the TEM image in Fig. 2a. The crystalline structure of the nanocomposite was then analyzed by X-ray diffraction (XRD). The XRD pattern of Fe@Arc (Fig. 2b) displayed a broad, diffuse halo, indicative of an amorphous carbon matrix, and notably, it was devoid of any distinct crystalline peaks corresponding to metallic iron or iron oxides. This suggested that the iron species were highly dispersed within the carbon framework. Furthermore, the elemental composition and spatial distribution were investigated using energy-dispersive X-ray spectroscopy (EDS) mapping (Fig. 2c and d). Results confirmed that the constituent elements—carbon (C), nitrogen (N), and iron (Fe)—were homogeneously distributed throughout the nanostructure, validating the successful formation of the composite. The drug loading capacity of Fe@Arc was quantified using high-performance liquid chromatography (HPLC), which determined an encapsulation efficiency of 10.9% ± 1.1% (w/w). Subsequently, to evaluate its potential as a delivery vehicle, the drug release profile of arctigenin from Fe@Arc was investigated under simulated physiological conditions in PBS.

To explore the bonding configurations of Fe and N in Fe@Arc, X-ray photoelectron spectroscopy (XPS) was employed. The high-resolution XPS spectrum revealed two distinct peaks in the Fe 2p region (Fig. 2e), corresponding to the Fe^3+^ (723.7 eV) and Fe^2+^ (710.1 eV) oxidation states. For the N 1s spectrum of Fe@Arc, deconvolution yielded two peaks, attributed to pyrrolic-N and graphitic-N (Fig. 2f). Similarly, the C 1s spectrum of Fe@Arc was split into three peaks, assigned to the C–C, C–N, and C=C bonds (Fig. 2g).

### 3.2 Catalytic activity

**Fig. 3:**
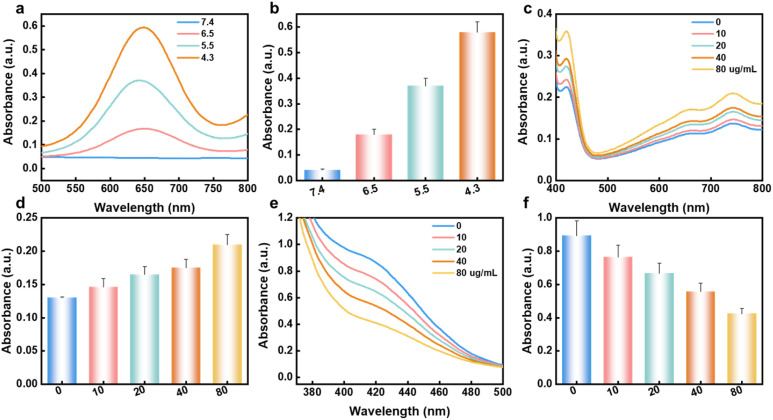
(a) and (b) UV-vis spectra of TMB treated with Fe@Arc plus H_2_O_2_ at different pH. (c) and (d) UV-vis spectra of ABTS treated with Fe@Arc plus H_2_O_2_ at different concentrations. (e) and (f) UV-vis spectra of DTNB treated with Fe@Arc plus GSH at varying concentrations.

We conducted a comprehensive assessment of the enzymatic activities of Fe@Arc, with special attention paid to its peroxidase (POD)-like and glutathione oxidase (GSHOX)-like activity. For quantifying the POD-mimicking activity of Fe@Arc, 3,3′,5,5′-tetramethylbenzidine (TMB) was employed as a chromogenic substrate. When Fe@Arc was incubated with H_2_O_2_ (Fig. 3a, b, S2 and 3), a rapid color shift of TMB was detected under acidic pH conditions (4.3–6.5), while no such change occurred at neutral pH (7.4). To further confirm the production of hydroxyl radicals (˙OH), 2,2′-azino-bis(3-ethylbenzothiazoline-6-sulfonic acid) diammonium salt (ABTS) was used as a detection probe. As illustrated in Fig. 3c and d, in the presence of H_2_O_2_, Fe@Arc induced a notable rise in the absorbance of ABTS, further validating its outstanding POD-like catalytic performance.

For evaluating the GSHOX-mimicking activity of Fe@Arc, 5,5′-dithiobis-(2-nitrobenzoic acid) (DTNB) was used as a reporter reagent. The absorbance at 412 nm (a characteristic signal of DTNB upon reacting with free thiols) declined sharply in an Fe@Arc concentration-dependent manner, which proved Fe@Arc's strong capacity to deplete glutathione (GSH) (Fig. 3e and f). As shown in Fig. S4, Arc can be effectively released from Fe@Arc in the presence of GSH.

### 3.3 *In vitro* antitumor effect

**Fig. 4:**
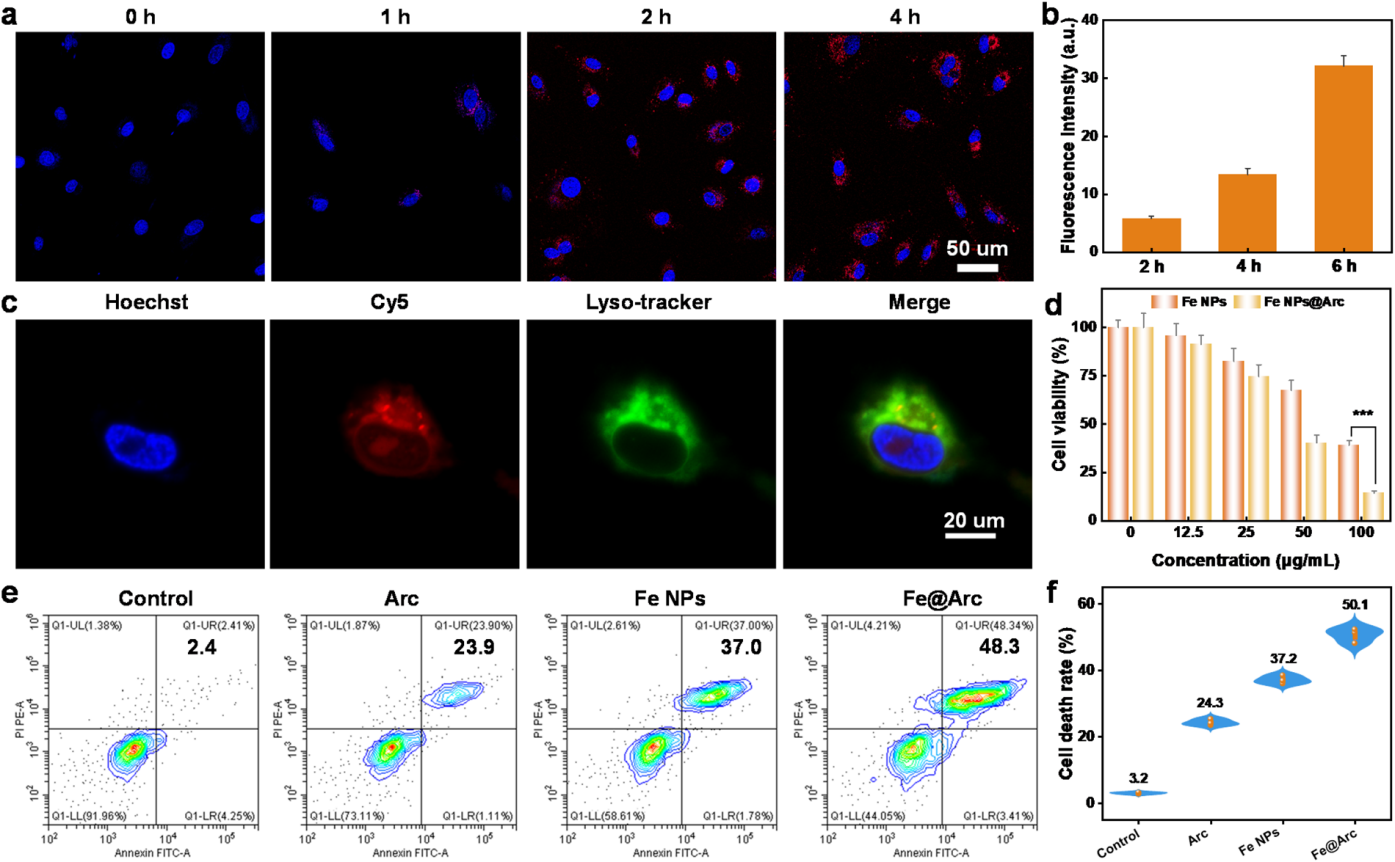
(a) Confocal laser scanning microscopy (CLSM) images of A549 cells incubated with Cy5-labeled Fe@Arc for different time periods at 37 °C. (b) Quantitative assessment of the cellular uptake efficiency of Cy5-labeled Fe@Arc. (c) Lysosomal colocalization analysis of A549 cells following treatment with Cy5-labeled Fe@Arc. (d) Relative viability of A549 cells after exposure to Fe@Arc at various concentrations. (e) Cell apoptosis analysis of A549 cells treated with different groups: control (PBS), free arctigenin (Arc), Fe nanoparticles (Fe NPs), and Fe@Arc. (f) Quantitative analysis of cell death rate based on flow cytometry results. All treatments were performed at an equivalent concentration corresponding to 50 µg mL^−1^ Fe@Arc. Data are expressed as mean ± SEM of three independent experiments.

To evaluate the intervention effect, the cells were treated with Fe@Arc nanozyme at a concentration of 50 µg mL^−1^. The concentrations of the components in the control group were calculated according to the constituents of Fe@Arc. To confirm the intracellular uptake of Fe@Arc, the fluorescent probe Cy5 was incorporated for confocal laser scanning microscopy (CLSM) imaging analysis. As presented in Fig. 4a and b, a distinct fluorescent signal was detected in A549 cells, confirming the successful internalization of the nanozyme. In this work, we also assessed the lysosomal escape capability of Fe@Arc in A549 cells. Our data revealed that the red fluorescent signal of Fe@Arc did not colocalize with the green fluorescent signal of lysosomes—this separation facilitated a more efficient intracellular delivery of the nanozyme (Fig. 4c and S5). The quantitative analysis revealed a Pearson's colocalization coefficient of 0.32019911. This result indicated a relatively low degree of colocalization between Fe@Arc and lysosomes. To evaluate the potential tumor-inhibiting effect of Fe@Arc, we first determined its cytotoxicity against A549 cells at various concentrations using the CCK-8 assay. Results from the CCK-8 assay demonstrated that Fe@Arc could effectively suppress tumor cell proliferation, and this inhibitory effect exhibited a clear concentration dependence (Fig. 4d). The IC_50_ value for the cell viability of arctigenin is 51.2 µM (Fig. S6). Additionally, the cytotoxicity of the drug-loaded nanozyme was visualized *via* live/dead staining, which was performed using the Annexin V-FITC/PI apoptosis detection kit (Yeasen, cat# 40302ES60). Flow cytometry analysis demonstrated that Fe@Arc treatment exerted a more potent tumor cell killing effect relative to control groups (Fig. 4e and f). It should be noted that the concentration of free arctigenin (Arc) used in the experiment was identical to that of the Arc encapsulated in Fe@Arc. Collectively, these results confirmed that Fe@Arc can efficiently induce tumor cell death.

### 3.4 Induction of cell ferroptosis

**Fig. 5:**
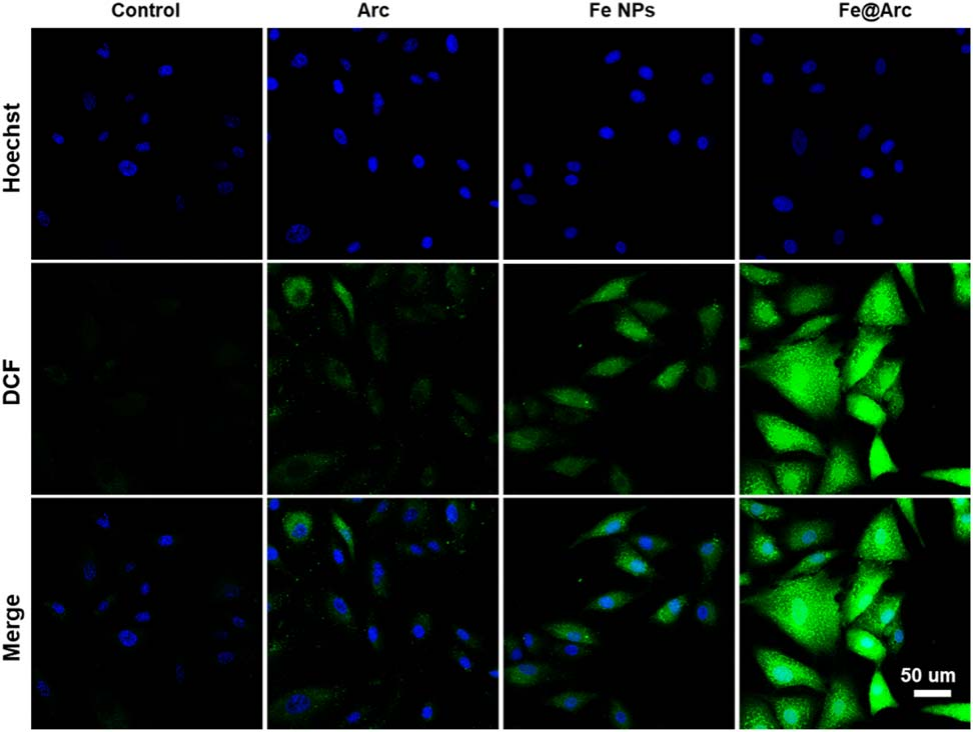
Confocal images of DCF in tumor cells scale bar = 50 µm.

**Fig. 6:**
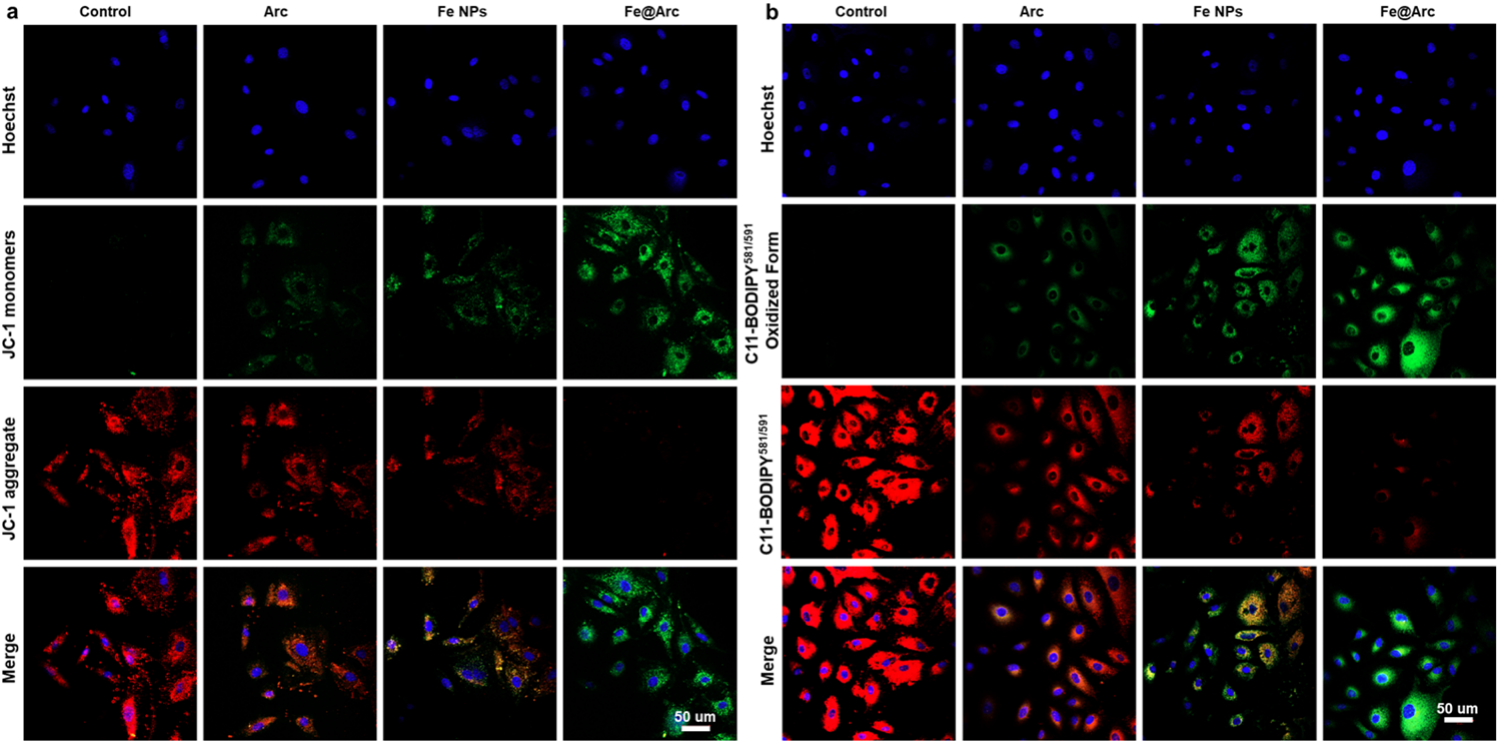
CLSM fluorescence images obtained using (a) JC-1 and (b) C11 BODIPY^581/589^. All treatments were performed at an equivalent concentration corresponding to 50 µg mL^−1^ Fe@Arc scale bar = 50 µm.

**Fig. 7:**
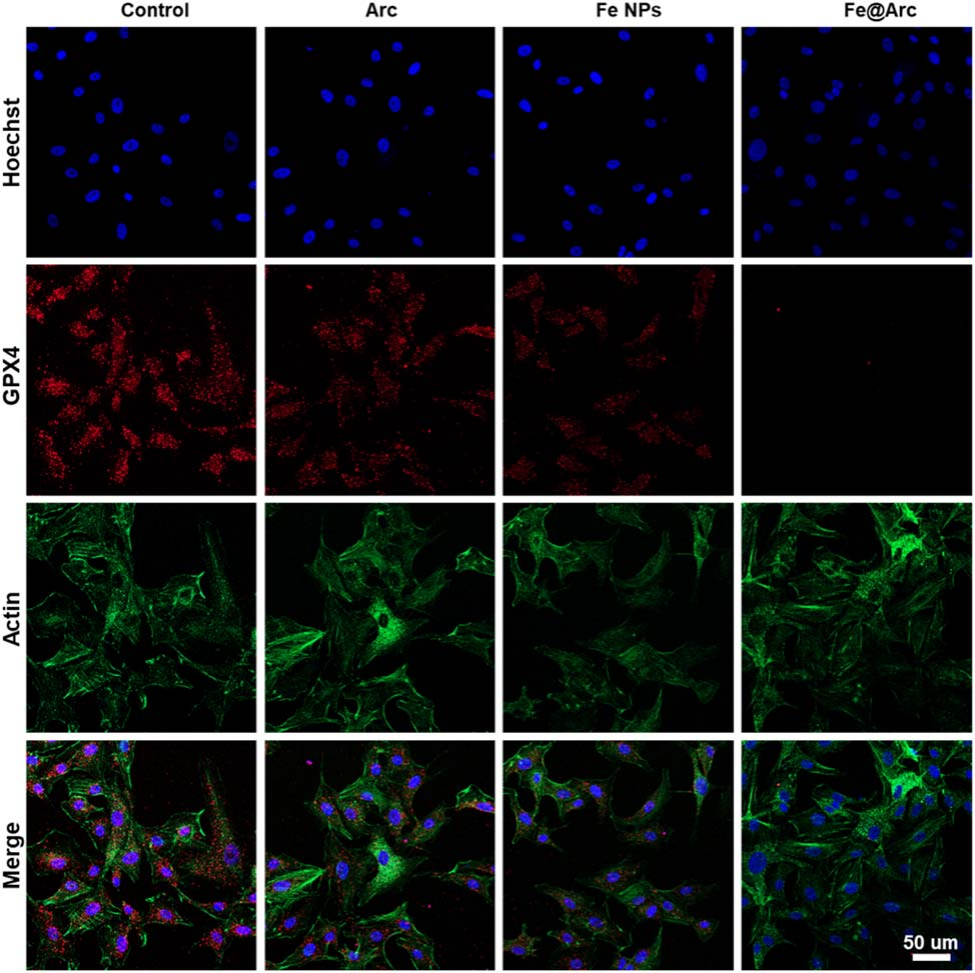
Confocal images of the GPX4 expression in tumor cells. All treatments were performed at an equivalent concentration corresponding to 50 µg mL^−1^ Fe@Arc scale bar = 50 µm.

To validate whether the Fe@Arc nanoplatform could effectively trigger ferroptosis, we conducted a series of *in vitro* assays. As shown in Fig. 5 and S7, treatment with Fe@Arc resulted in a significant elevation in intracellular ROS levels in tumor cells, a critical upstream event for ferroptosis. Furthermore, to assess mitochondrial dysfunction, a hallmark of ferroptosis, we employed the JC-1 probe to monitor mitochondrial membrane potential (Δ*Ψ*_m_). The results shown in Fig. 6a reveal a marked increase in green fluorescence intensity in the Fe@Arc-treated group compared to that in the control group, indicating a depolarization of the mitochondrial membrane. To directly measure the hallmark accumulation of lipid peroxides, we utilized the fluorescent indicator C11-BODIPY^581/589^.^28^ Upon oxidation, this probe shifts its emission from red to green. Consistent with ferroptosis induction, Fe@Arc treatment greatly amplified the green fluorescence signal while concurrently diminishing the red signal (Fig. 6b), providing a clear evidence of extensive lipid peroxidation (LPO). Finally, an immunofluorescence assay was performed to investigate the key regulatory protein, glutathione peroxidase 4 (GPX4).^29,30^ In addition, we have performed qPCR and ELISA analyses of key ferroptosis markers. As shown in Fig. S8, the GPX4 mRNA level was significantly downregulated following the Fe@Arc treatment. Furthermore, we assessed additional ferroptosis markers, including malondialdehyde (MDA) and 4-hydroxynonenal (4-HNE). The Fe@Arc treatment significantly elevated MDA and 4-HNE levels in tumor cells (Fig. S9). Fig. 7 confirms that the Fe@Arc treatment led to a profound suppression in the GPX4 expression. Collectively, these findings provided a compelling evidence that Fe@Arc effectively induced tumor cell ferroptosis through a multi-faceted mechanism involving ROS amplification, mitochondrial damage, lipid peroxide accumulation, and GPX4 inhibition. Furthermore, we employed the ferroptosis inhibitor DFO to corroborate that Fe@Arc induced ferroptosis (Fig. S10). The experimental results demonstrated that the addition of DFO significantly reduced cytotoxicity, indicating that Fe@Arc exerts its tumor suppressive effects primarily through the induction of ferroptosis.

### 3.5 *In vivo* anticancer therapy and microenvironment regulation

**Fig. 8:**
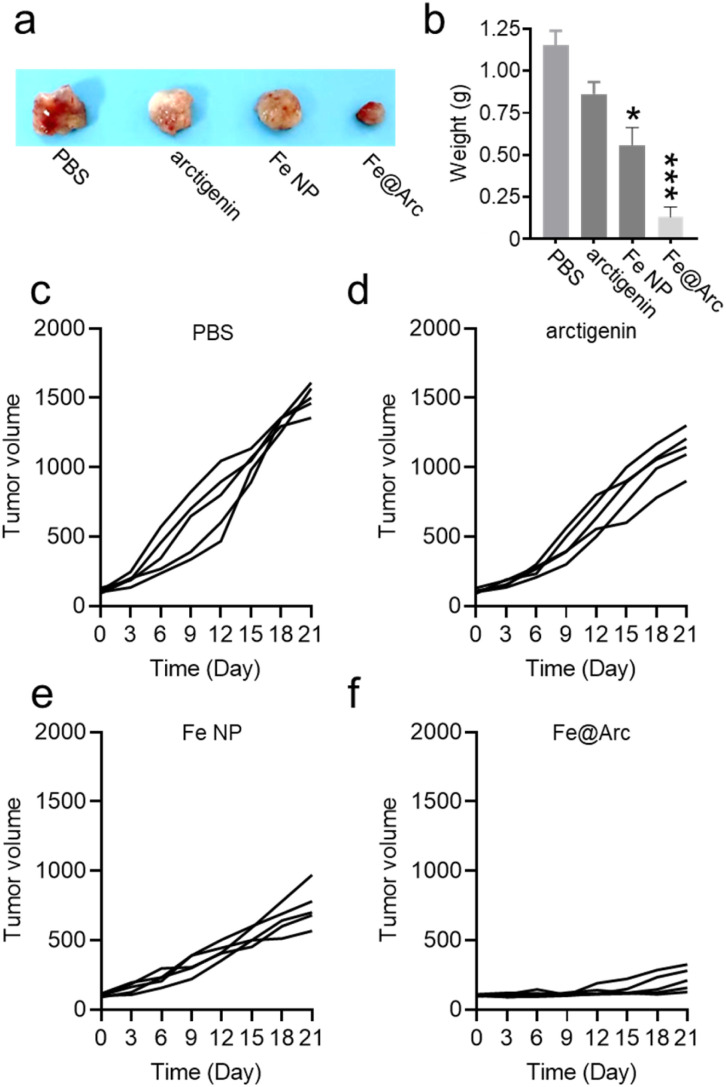
(a) Images of the excised tumors of mouse in different treatment groups. (b) Weight of the excised tumors of different groups. (c–f) Tumor volume curve of each treatment group. Nude mice bearing tumors were treated *via* tail vein injections of four different agents: PBS (control), free arctigenin, Fe nanoparticles (Fe NPs), and Fe@Arc, with the dose of each agent standardized to 2 mg of arctigenin per kg of mouse body weight (Fe@Arc nanozyme containing 2 mg of arctigenin, and Fe NPs containing an equivalent amount of Fe to that in Fe@Arc). The data are presented as mean ± SEM (*n* = 5). (*p < 0.05, **p < 0.01, ***p < 0.001).

Inspired by the outstanding *in vitro* therapeutic performance of Fe@Arc, we subsequently investigated its *in vivo* anticancer efficacy to further assess its therapeutic potential. The *in vivo* tumor-inhibiting efficiency was evaluated using an A549 tumor-bearing nude mouse model. Nude mice bearing tumors were treated *via* tail vein injection of four different agents: PBS (control), free arctigenin, Fe nanoparticles (Fe NPs), and Fe@Arc with the dose of each agent standardized to 2 mg of arctigenin per kg of mouse body weight (Fe@Arc nanozyme containing 2 mg of arctigenin, and Fe NPs containing an equivalent amount of Fe to that in Fe@Arc). Drug administration was performed every other day, with a total of three doses given on day 0, day 3, and day 6. Over the 21 day observation period, tumors in the PBS control group exhibited a rapid growth trend. As presented in Fig. 8a–f, Fe@Arc achieved a significantly high effective tumor growth inhibition compared with free arctigenin. These results collectively indicate that Fe@Arc possesses excellent therapeutic efficacy for cancer treatment. Although the encapsulation efficiency of Fe@Arc was approximately 11%, the current formulation successfully achieved a significant therapeutic effect in the tumor model. This suggests that the current drug loading was sufficient to trigger the desired ferroptosis pathway. The moderate encapsulation efficiency was likely due to the hydrophilic nature of the drug. In our future studies, we intend to explore the addition of co-solvents or other solubilizing agents to improve the encapsulation efficiency. Despite this limitation, the prominent anti-tumor efficacy observed confirms the clinical potential of the synthesized complex.

## 4 Conclusions

In conclusion, this study reports the rational design, successful synthesis, and comprehensive evaluation of a novel arctigenin-based nanoplatform, designated Fe@Arc, for the targeted therapy of lung cancer. Our findings robustly demonstrate that this nanosystem effectively harnesses a potent multi-modal synergistic interaction between its core components. The iron core catalyzes the Fenton reaction, depleting intracellular GSH and inactivating the key ferroptosis-inhibiting enzyme, GPX4. Concurrently, the loaded arctigenin acts as a chemosensitizer, amplifying intracellular ROS levels. This dual-pronged assault culminates in the robust induction of ferroptosis, leading to a significant anti-tumor efficacy *in vivo*. The therapeutic strategy presented herein, which centers on ferroptosis as a pivotal molecular target, offers a promising and innovative approach to overcome the limitations of conventional therapies. By validating the feasibility, anti-tumor potency, and favorable biosafety profile of Fe@Arc, this work not only introduces a compelling candidate for further preclinical development in lung cancer intervention but also establishes a solid methodological foundation for future research on nanozyme-mediated ferroptosis therapy. It opens new avenues for the future design and clinical translation of next-generation ferroptosis-targeted cancer nanomedicines, particularly those integrating bioactive natural products.

## Author contributions

Y. J.: methodology, conceptualization, data curation, writing – original draft. C. H.: methodology, validation, writing – original draft. Z. G.: writing – original draft. H. L.: writing – original draft. H. G.: formal analysis, validation, writing – review and editing. P. W.: writing – review and editing. C. D.: supervision, writing – review and editing. J. L.: formal analysis, writing – original draft. X. Y.: writing – review and editing. C. L.: writing – review and editing. Y. Z.: funding acquisition, supervision, writing – review and editing.

## Conflicts of interest

The authors declare that the research was conducted in the absence of any commercial or financial relationships that could be construed as a potential conflict of interest.

## Supplementary Material

RA-016-D6RA00383D-s001

## Data Availability

The datasets generated and/or analyzed during the current study are available from the corresponding author on reasonable request. Supplementary information (SI) is available. See DOI: https://doi.org/10.1039/d6ra00383d.
