# Supplementary material for: A Natural product-based composite nanozyme synergistically induces ferroptosis for lung cancer therapy
Source: RSC Adv. 2026 May 26;16(31):28583–91. doi: 10.1039/d6ra00383d (PMC13213561; doi:10.1039/d6ra00383d)
Supplement: RA-016-D6RA00383D-s001 [file RA-016-D6RA00383D-s001.pdf]

This journal is © The Royal Society of Chemistry 2026

1 Supplementary Figure

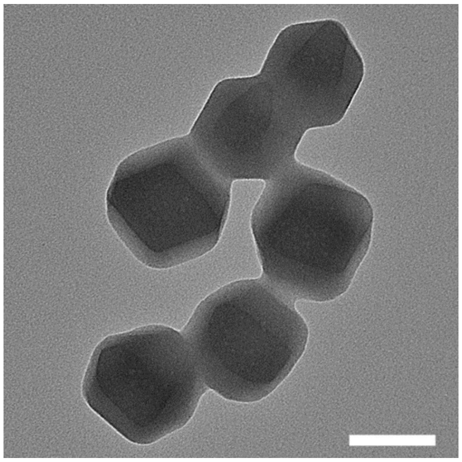

Supplementary Figure S1. TEM image of the ZIF-8. Scale bar: 100 nm.

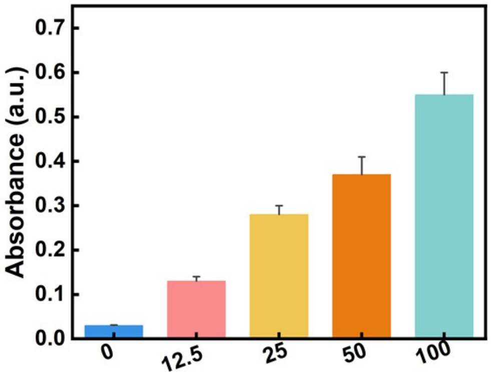

Supplementary Figure S2. UV-Vis spectra of different concentrations of Fe@Arc treated with TMB plus H_2O_2.

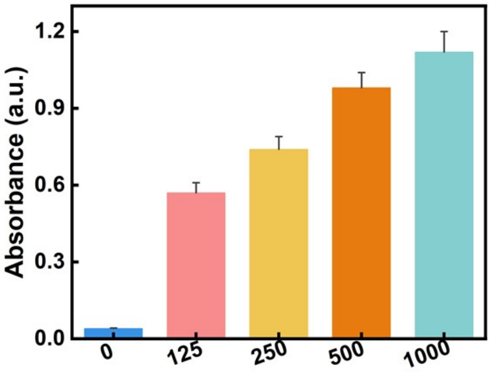

Supplementary Figure S3. UV-Vis spectra of different concentrations of H_2O_2 treated with TMB plus Fe@Arc.

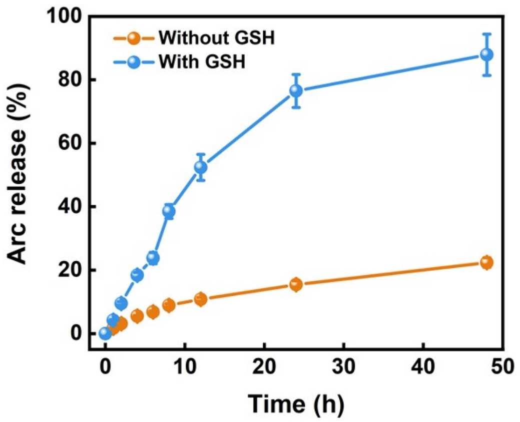

Supplementary Figure S4. The Arc release curves in the presence of GSH.

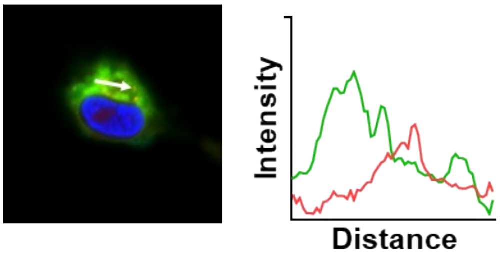

Supplementary Figure S5. Lysosomal colocalization analysis of A549 cells.

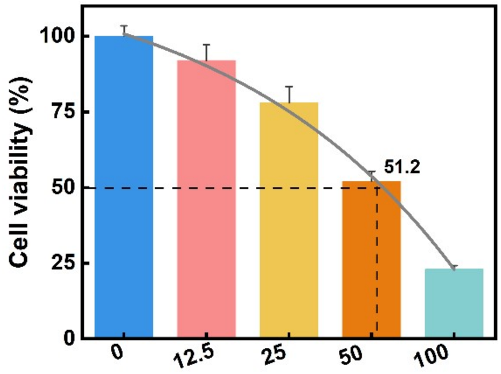

Supplementary Figure S6. The cell viability and IC_{50} of Arctigenin.

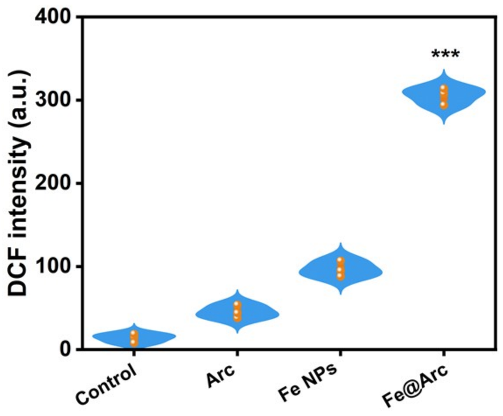

Supplementary Figure S7. Quantitative analysis of cell death rate based on confocal image's results.

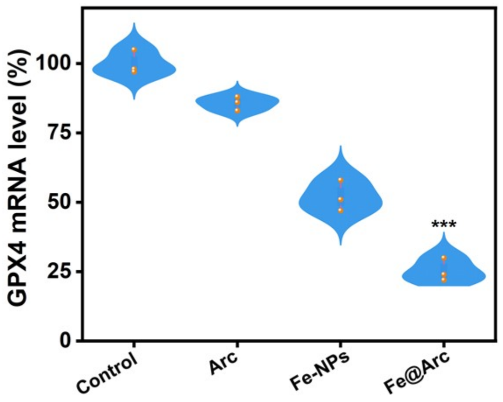

Supplementary Figure S8. The GPX4 mRNA level of cancer cells following varying treatments.

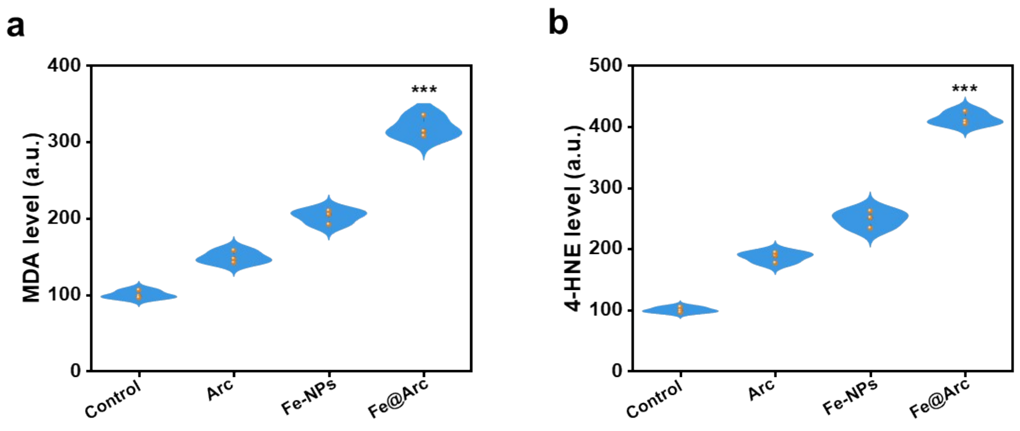

Supplementary Figure S9. (a) MDA and (b) 4-HNE levels in tumor cells following various treatments.

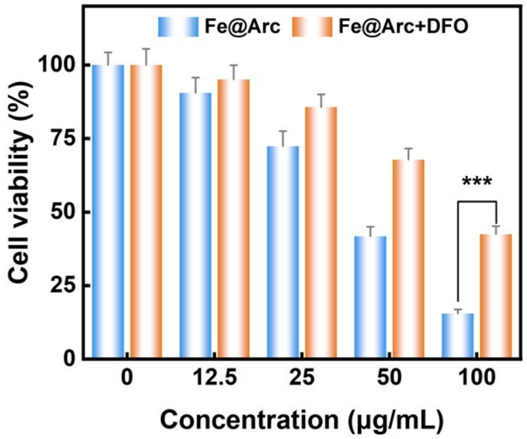

Supplementary Figure S10. The cell viability of Fe@Arc in the presence of DFO. The data are presented as mean ± SEM (n=5). (*** $p < 0.001$)
